# Preface to supplement, CMA4CH 2010: Multivariate Analysis and Chemometry to Cultural Heritage and Environment

**DOI:** 10.1186/1752-153X-6-S2-I1

**Published:** 2012-05-02

**Authors:** Giovanni Visco

**Affiliations:** 1 Universitya "La Sapienza" Rome, Italy

## 

This supplement contains an accurate selection of papers based on the research presented at the 3^rd^ edition of the International Meeting CMA4CH with a balance in the three main topics.

The meeting was held in Taormina on 26-29 September 2010 and the main aim was to favour the meeting of all professional figures involved in the protection of cultural heritage (CH) as well as of the environment. In reality the two topics are very close as a healthy environment ensures a longer life of our CH and, as a consequence, the latter can be considered an “indicator” of a safe environment taking into account that, in most cases, pollution plays a similar role on objects and humans.

Due to the big mole, and overall to the heterogeneity, of data coming from a significant monitoring of the environment or from analyses aimed to the conservation of CH, a classical data treatment treats results hard and most of the information can be lost. This is the reason that pushed us to join chemometry [1] and multivariate [2] analyses with the previous two topics.

Even if the best field of research were to include all the three topics, this is not so common; anyway the importance of multivariate techniques is acknowledged with an increasing trend and really many research groups include at least one expert on the subject. Multivariate techniques born to satisfy industrial needs and applications on environment and CH are relatively recent; so for many chemometricians our meetings can be a new, very interesting approach.

Research presented at our meeting generally treat environment or CH coupled to multivariate or chemometric [3,4] analyses, but few of them lack the statistical treatment and were presented looking for a cooperation with chemometricians; on the other hand some pure chemometricians presented communications treating only the theoretical aspects in order to evidence the sure improvement of their data treatment with respect to the classical one.

All papers here published passed a three, serial, refereeing; the first to check consistency and quality balance, the second to check chemical/physical aspects, the third to check multivariate and chemometry content [5]. Although slow, this method ensured an increasing quality passing among the three steps.

Where possible all papers were revised in order to facilitate understanding for readers with no experience in the chemometrics field [6].

**Figure 1:**
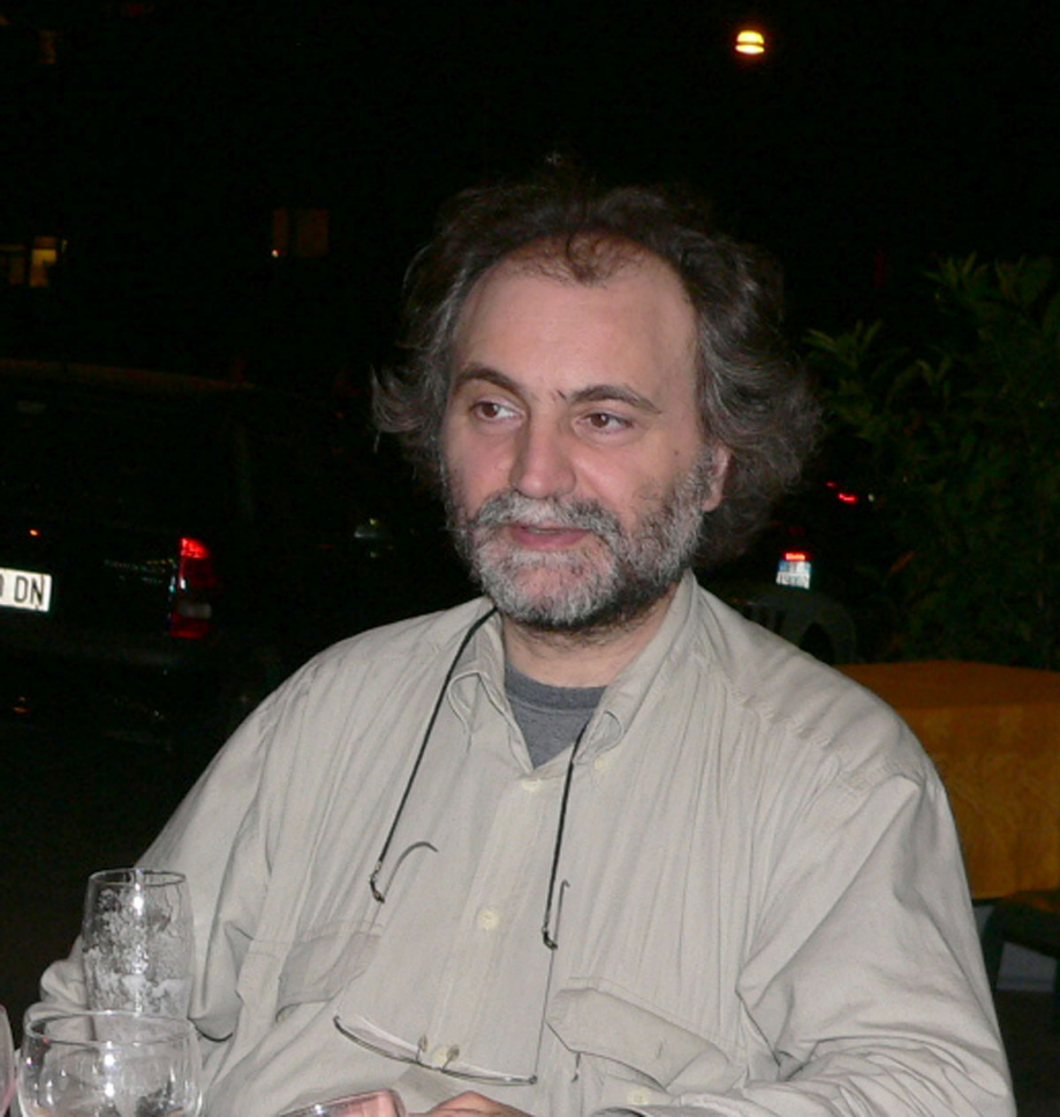
The Coordinator, Dr. Giovanni Visco, Appointed Professor for Chemometrics at Rome University

First of all I (fig. 1) want to thank all the authors for their great patience in following the rules for the non-typical refereeing method, this allowed us to obtain a very homogeneous set of papers and, I hope, papers of a high scientific content.

Very warm thanks are due to the referees, especially to Prof. JB Ghasemi, Prof. AM Salvi, Dr F Marini, Dr P Ielpo, Dr. SH Plattner and of course to Dr MP Sammartino, for their hard work under pressure.

Last, but not least, I warmly thank the scientific committee's members that spent some of their time, diverting it from the didactic and research activities, aiming to attain a full success for the conference. In particular Prof. Richard Brereton gave a big contribution looking for “prizes” and help with journal(s) contacts.

I look forward to seeing all of you and a lot of your friends and colleagues in the next Meeting that will be held in the beautiful Rome, surely the richest Occidental city of Cultural Heritage on the end of May 2012.
